# Breaking new ground: first report of integrating clinical, hematobiochemical, sonographic, and pathological findings in dromedary camels (*Camelus dromedarius*) with hepatic fibrosis

**DOI:** 10.3389/fvets.2025.1639628

**Published:** 2025-07-09

**Authors:** Mohamed Tharwat, Hazem M. M. Elmoghazy, Mohie Haridy

**Affiliations:** ^1^Department of Clinical Sciences, College of Veterinary Medicine, Qassim University, Buraidah, Saudi Arabia; ^2^University Veterinary Hospital, Qassim University, Buraidah, Saudi Arabia; ^3^Veterinary Teaching Hospital, Faculty of Veterinary Medicine, Benha University, Benha, Egypt; ^4^Department of Pathology and Laboratory Diagnosis, College of Veterinary Medicine, Qassim University, Buraidah, Saudi Arabia

**Keywords:** camels, fibrosis, liver, pathology, ultrasound

## Abstract

**Introduction:**

Hepatic fibrosis is a progressive liver disorder that can lead to significant morbidity in animals, yet its characteristics remain poorly described in dromedary camels (*Camelus dromedarius*). This study aims to provide a comprehensive characterization of the clinical, hematobiochemical, ultrasonographic, and histopathological features associated with hepatic fibrosis in dromedaries.

**Methods:**

Sixteen female camels presenting with clinical signs of inappetence, weight loss, and discolored urine were evaluated and compared with eleven clinically healthy controls. All animals underwent clinical examination, hematological and biochemical analyses, ultrasonographic imaging of the liver, and histopathological assessment of liver tissue samples.

**Results:**

Affected camels exhibited clinical indicators of chronic illness, including leukocytosis, neutrophilia, anemia, elevated alkaline phosphatase levels, and hyperglobulinemia. Ultrasonographic findings included increased hepatic echogenicity, nodular parenchymal patterns, indistinct hepatic vasculature, and the presence of peritoneal effusion. Histopathological examination revealed portal and bridging fibrosis, biliary hyperplasia, hepatocellular atrophy, Kupffer cell hyperplasia, and inflammatory infiltration. Parasitic structures were identified in some liver samples, suggesting a possible etiological factor.

**Discussion:**

The study provides the first detailed, multimodal diagnostic profile of hepatic fibrosis in dromedary camels. The combination of clinical signs, laboratory abnormalities, imaging features, and histopathological findings supports the utility of an integrated diagnostic approach. The identification of parasitic structures in some cases warrants further investigation into potential infectious etiologies.

## 1 Introduction

Liver fibrosis in humans is characterized by the excessive accumulation of extracellular matrix proteins, such as collagen, in response to chronic liver injury. This scarring disrupts normal liver architecture and function. Major causes include chronic viral infections, alcohol use, and nonalcoholic steatohepatitis (1, 2). If untreated, fibrosis can progress to cirrhosis, liver failure, and portal hypertension, significantly increasing the risk of hepatocellular carcinoma. Early detection is critical, as fibrosis is often asymptomatic in its early stages. Recent advances have highlighted the central role of hepatic stellate cells in fibrogenesis, paving the way for targeted antifibrotic therapies (3).

In veterinary medicine, hepatic fibrosis can occur in cattle due to chronic infections such as fasciolosis, caused by *Fasciola hepatica*. Affected animals may exhibit reduced appetite, decreased milk yield, weight loss, anemia, and hypoalbuminemia (4). It is also commonly observed in dairy cows with fatty liver disease (5). In horses, hepatic fibrosis often results from chronic liver diseases, including infection with equine hepacivirus (EqHV), a virus analogous to hepatitis C in humans. Persistent EqHV infection may lead to cirrhosis (6). Clinical signs in horses include anorexia, weight loss, jaundice, behavioral changes, and depression (7). Theiler’s disease, an acute hepatitis associated with equine-origin biologics, has also been implicated in liver failure in horses (8).

Hepatic diseases are relatively common in dromedary camels (9). In one abattoir study, 44 camel livers with pathological changes were examined; lesions included fatty infiltration (47.7%), cirrhosis and hepatitis (27.2%), hepatocellular necrosis (18.1%), and cholestasis with biliary hyperplasia (6.8%) (43). Another large-scale survey of 150 livers found hepatic lesions in 40 cases (26.7%), including hydatid cysts (65%), cirrhosis (10%), fatty infiltration (12.5%), glycogen deposition (2.5%), cholangitis (2.8%), cholangiohepatitis (5%), calcified hydatid cysts (2.5%), liver abscesses (2.5%), and lipofuscin pigment accumulation (17.5%) (44). Reported cirrhosis rates in these studies were 27.2 and 10%, respectively.

In camels, liver disease is often underdiagnosed *ante mortem* due to vague or nonspecific clinical signs. Moreover, hematology and serum biochemistry alone may be insufficient for definitive diagnosis (9). Ultrasonography is a valuable diagnostic tool for evaluating liver parenchyma and vasculature in both healthy and diseased camels (9–12). Ultrasound-guided hepatic biopsy is a safe, rapid, and cost-effective method for histopathological evaluation when performed correctly (13). Additionally, real-time ultrasound-guided portocentesis has been shown to be an accurate technique for portal vein blood sampling in camels (14). Liver biopsy remains the gold standard for assessing hepatic histopathology, offering critical insights into the degree of fibrosis, necrosis, parenchymal integrity, bile duct architecture, and deposition of storage materials and minerals (45).

The study aims to describe the clinical, hematobiochemical, ultrasonographic, and pathological findings associated with liver fibrosis in dromedary camels (*Camelus dromedarius*).

## 2 Materials and methods

### 2.1 Camels and clinical examinations

Between November 2023 and May 2025, 16 female dromedary camels (*Camelus dromedarius*), aged 10 to 18 years, were examined at Qassim University Veterinary Hospital. Camels included in this study were selected based on the presence of inappetence, and progressive weight loss, with reported disease durations ranging from 3 to 9 months. Prior treatments included antibiotics, anti-inflammatories, appetite stimulants, and multivitamin supplements.

Each camel underwent a comprehensive examination, including the assessment of respiratory rate, pulse, rectal temperature, mucosal inspection, and auscultation of the thorax and gastrointestinal tract. Eleven healthy camels were selected as controls based on clinical, sonographic, hematological, and biochemical findings. All procedures adhered to ethical guidelines approved by the Ethics Committee for Animal Use at Qassim University (Buraydah, Saudi Arabia) and followed the Guide for the Care and Use of Agricultural Animals in Research and Teaching (15).

### 2.2 Determination of hematobiochemical parameters

Jugular puncture was performed on both diseased and healthy camels to collect two blood samples. The first sample, drawn into EDTA tubes, was used to determine hemogram (erythrocyte count, erythrocyte indices, hematocrit, and hemoglobin concentration) and leukogram (white blood cell count and differential). The second sample, collected in plain tubes, was used to separate serum for measuring concentrations of albumin, amylase, blood urea nitrogen (BUN), calcium, creatinine, globulins, glucose, inorganic phosphorus, potassium, sodium, total bilirubin, total protein, and the activity of alanine aminotransferase (ALT) and alkaline phosphatase (ALP).

### 2.3 Hepatic ultrasonography

For hepatic sonography, the right side of the abdomen—from the 5th intercostal space to the right flank—was clipped and shaved. The transducer was coated with transmission gel, and the liver was examined starting at the right paralumbar fossa and progressing cranially to the 5th intercostal space. A 3.5 MHz sector transducer (Sonoscape Medical Corp., China) was used for all examinations. The ultrasound machine was adjusted with a depth setting of 10–20 cm, gain optimized between 50 and 90%, and a focal zone set at the level of the liver. The transducer was positioned perpendicularly and obliquely to the abdominal wall to obtain both longitudinal and transverse images. The evaluation included hepatic echotexture, visualization of the hepatic and portal veins, and assessment of both diaphragmatic and visceral surfaces (12). Additionally, sonographic examination of thoracic and abdominal organs—including the heart, major vessels, lungs, pleura, stomach compartments, intestines, liver, peritoneum, kidneys, and urinary bladder—was conducted using both transcutaneous and rectal approaches (16).

### 2.4 Ultrasound-guided hepatic biopsy and histopathological work

After obtaining written approval from the owners, hepatic lesions were sampled using a free-hand ultrasound-guided aspiration technique with a 14G × 170 mm spinal biopsy needle (Kurita Co., Ltd., Tokyo, Japan) as described by Tharwat et al. (13). The biopsy site was selected, clipped, shaved, and prepared non-septically. Two milliliters of Xylazine 2% (0.2 mg/kg BW) (Xylased, Bioveta, Czech Republic) were injected IV, followed by 10 mL of Procaine HCL 2% (Lidocaine Hydrochloride, Pharmaceutical Solutions Industry, Jeddah, Saudi Arabia) for local anesthesia. The needle was guided to the thickest part of the lesion, and a sample was collected. Biopsy samples were fixed in 10% neutral buffered formalin, embedded in paraffin wax, sectioned (3 μm), and stained with hematoxylin and eosin (H&E) and Masson’s Trichrome (17).

### 2.5 Statistics

Data are expressed as mean ± SD, with minimum and maximum values, and were analyzed using Student’s *t*-test (18). *p*-values ≤0.05 were considered statistically significant.

## 3 Results

**Figure 1:**
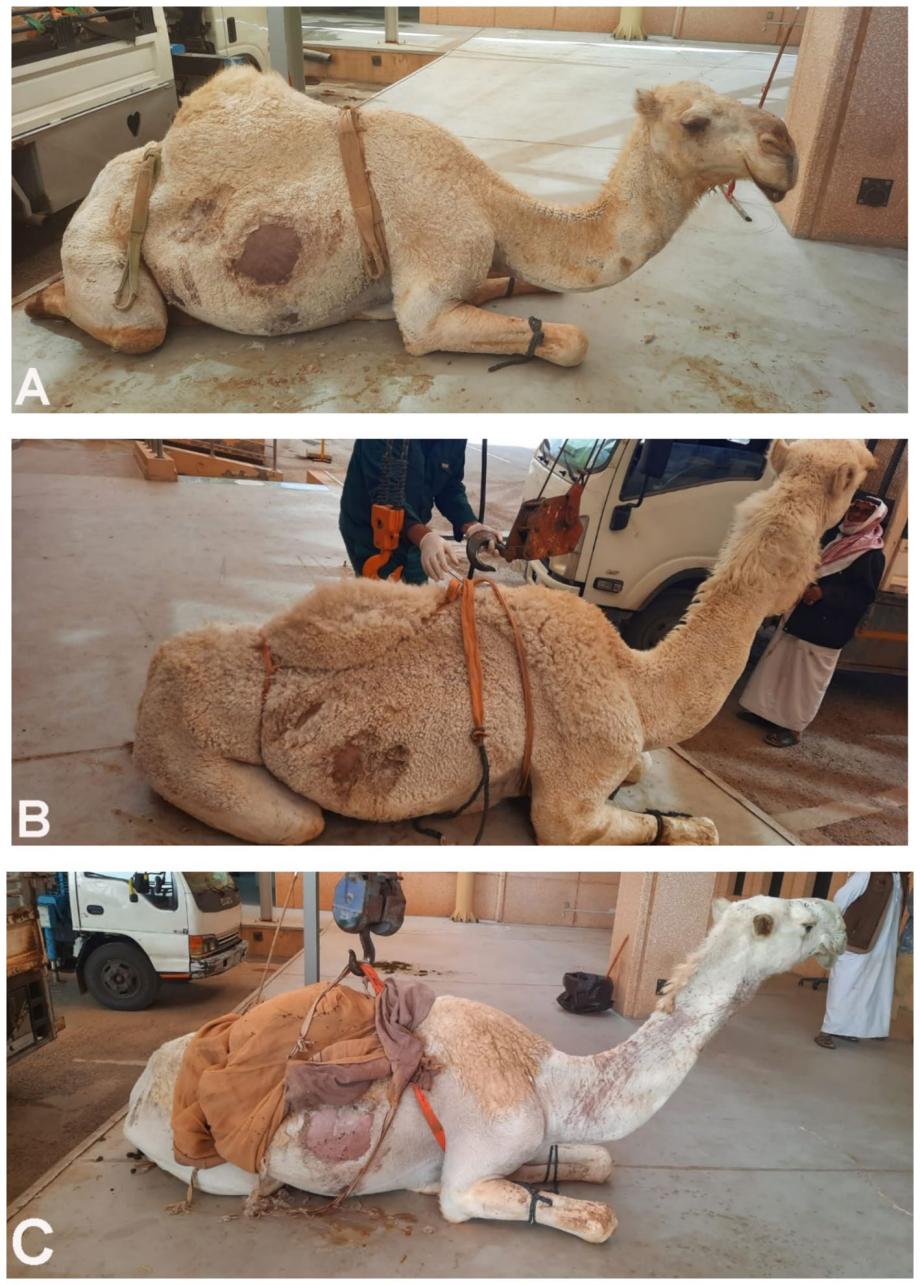
Dromedary camels diagnosed with liver fibrosis. Presenting complaints included loss of appetite and poor body condition for 3 months **(A)** and 6 months **(B)**. In the camel shown in image **C**, the main complaint was the passage of red-colored urine for 1 month; further renal ultrasonography revealed a right kidney abscess.

**Figure 2:**
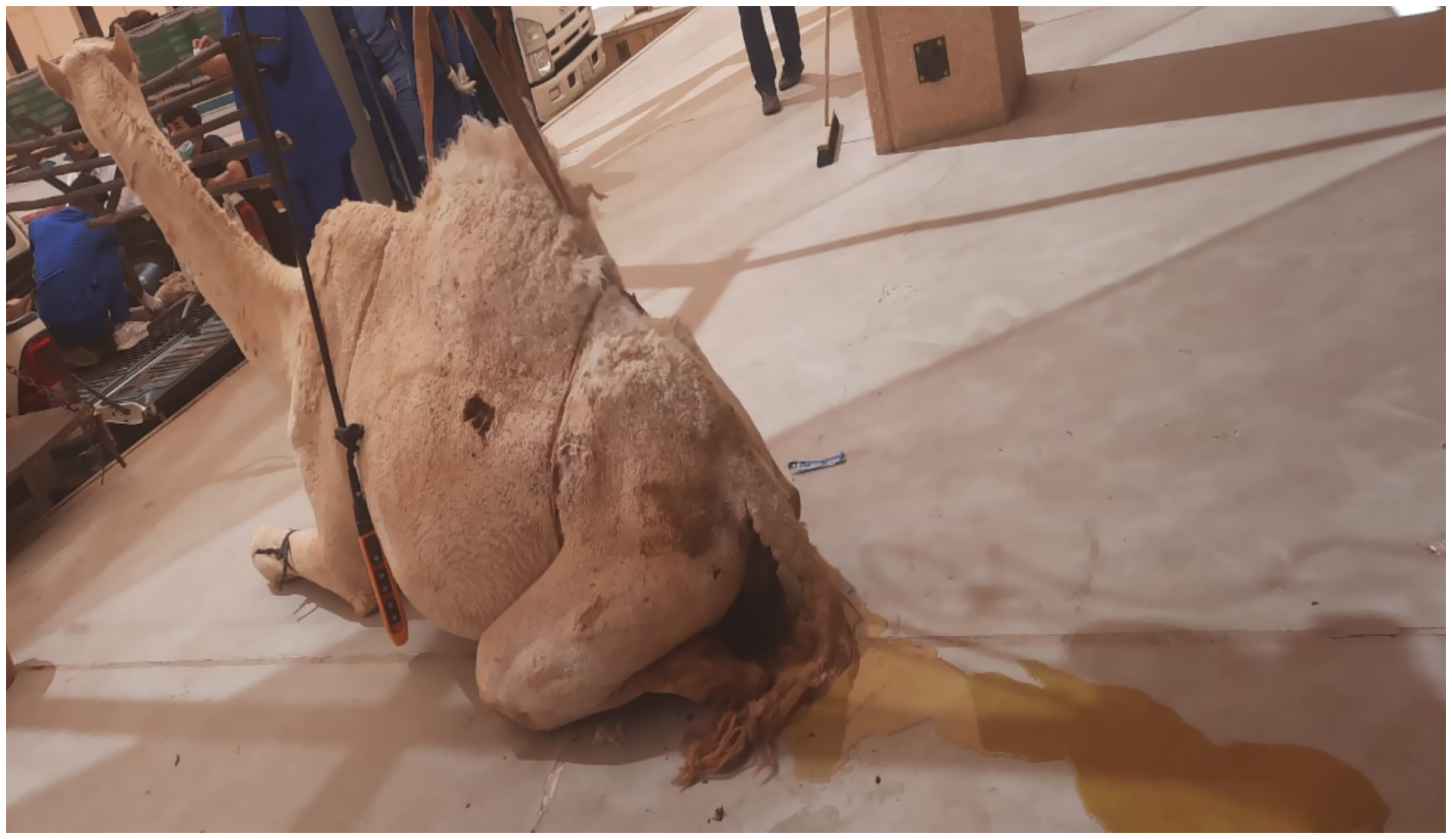
Deep yellow urine in a female dromedary camel with confirmed liver fibrosis.

In the 16 dromedary camels exhibiting clinical illness, common signs included inappetence, anorexia, and progressive weight loss over a period of 1 to 6 months. Rectal temperature, pulse, and respiratory rates were 35.0 ± 2.2°C, 40 ± 15/min, and 10 ± 4/min, respectively. Body condition scores ranged from 1.5 to 2.5 (1–5 scale). Mucous membranes were pale in all diseased camels, with one showing icterus. Abnormalities in rumen contractions and intestinal motility were observed in 11 (73.3%) and 10 (66.7%) of the camels, respectively. A concurrent hematuria was observed in only one camel owing to right kidney abscessation (Figure 1), and another presented with deep yellow urine (Figure 2).

Table 1 summarizes the hematobiochemical alterations in the 16 diseased camels compared to 11 healthy controls. Blood count abnormalities in diseased camels included anemia (*p* = 0.01), decreased hematocrit (*p* = 0.002), and increased mean corpuscular hemoglobin (*p* = 0.003). Leukogram changes were characterized by eosinophilia (*p* = 0.05), leukocytosis (39.0 ± 13.6 × 10^3^/μL vs. 13.2 ± 3.8 × 10^3^/μL, *p* < 0.0001), monocytosis (*p* = 0.0001), and neutrophilia (34.5 ± 12.6 × 10^3^/μL vs. 10.0 ± 3.7 × 10^3^/μL, *p* < 0.0001). Biochemical findings included elevated alkaline phosphatase (ALP; 216 ± 135 U/L vs. 37.8 ± 10.4 U/L, *p* = 0.0002), hyperglobulinemia (*p* < 0.0001), hyperphosphatemia (*p* = 0.01), hypocalcemia (*p* = 0.04), and increased total protein levels (*p* = 0.001). No significant differences were observed in other parameters such as alanine aminotransferase (ALT), albumin, and glucose (*p* > 0.05).

**Table 1:** Hematobiochemical alterations in dromedary camels with hepatic fibrosis (*n* = 16) compared to healthy controls (*n* = 11).

Parameters	Diseased (*n* = 16)			Controls (*n* = 11)			*p*
	Mean ± SD	Min	Max	Mean ± SD	Min	Max	
Leukocytes (10^3^/μL)	39.0 ± 13.6	9.1	68.3	13.2 ± 3.8	7.0	19.8	<0.0001
Lymphocyte (10^3^/μL)	1.6 ± 1.1	0.2	4.6	1.7 ± 0.9	0.5	3.5	0.7
Monocyte (10^3^/μL)	0.2 ± 0.1	0.1	0.4	0.1 ± 0.0	0.1	0.2	0.0001
Neutrophil (10^3^/μL)	34.5 ± 12.6	7.7	63.5	10.0 ± 3.7	4.3	15.5	<0.0001
Eosinophil (10^3^/μL)	2.6 ± 1.6	0.7	5.1	1.5 ± 1.0	0.3	3.7	0.05
Erythrocytes (10^6^/μL)	9.5 ± 1.7	6.9	12.2	11.0 ± 0.1	9.6	12.6	0.01
Hemoglobin (g/dL)	13.3 ± 2.7	9.1	19.8	13.8 ± 1.3	11.4	16.1	0.5
Hematocrit (%)	24.8 ± 3.5	17.9	30.1	29.2 ± 2.7	23.7	33.6	0.002
MCV (fL)	26.4 ± 1.7	24.0	29.0	26.8 ± 2.4	22.0	30.0	0.6
MCH (pg)	14.0 ± 1.2	12.6	16.3	12.6 ± 1.0	10.5	14.2	0.003
MCHC (g/dL)	53.3 ± 6.1	47.7	69.1	47.2 ± 1.5	45.3	50.1	0.003
Albumin (g/dL)	2.5 ± 0.6	1.6	3.4	7.3 ± 11.2	2.7	41.1	0.1
Alkaline phosphatase (U/L)	216 ± 135	69	436	37.8 ± 10.4	17	52	0.0002
ALT (U/L)	9 ± 5	5	17	12 ± 2	7	15	0.1
Amylase (U/L)	866 ± 288	601	1,688	992 ± 157	752	1,274	0.2
Total bilirubin (g/dL)	0.4 ± 0.4	0.2	1.8	0.3 ± 0.1	0.2	0.4	0.2
Blood urea nitrogen (g/dL)	2 ± 21	9	93	24 ± 17	6	71	0.8
Calcium (g/dL)	9.8 ± 0.5	8.6	10.5	10.2 ± 0.5	9.7	11	0.04
Phosphorus (g/dL)	8.0 ± 2.1	4.6	11.8	5.6 ± 2.2	2.8	9.4	0.01
Creatinine (g/dL)	1.6 ± 0.7	0.9	3.9	1.7 ± 0.4	1.2	2.5	0.7
Glucose (g/dL)	141 ± 45	55	248	158 ± 36	99	231	0.3
Sodium (mmol/L)	144 ± 4	139	153	148 ± 5	139	157	0.08
Potassium (mmol/L)	4.5 ± 0.6	3.9	5.9	4.7 ± 0.5	3.9	5.5	0.3
Total protein (g/dL)	8.2 ± 1.0	5.4	9.4	6.8 ± 0.8	5.8	7.8	0.001
Globulin (g/dL)	5.7 ± 1.2	2.6	7.5	2.9 ± 0.5	2.2	3.7	<0.0001

MCV, mean corpuscular volume; MCH, mean corpuscular hemoglobin; MCHC, mean corpuscular hemoglobin concentration; ALT, alanine aminotransferase.

**Figure 3:**
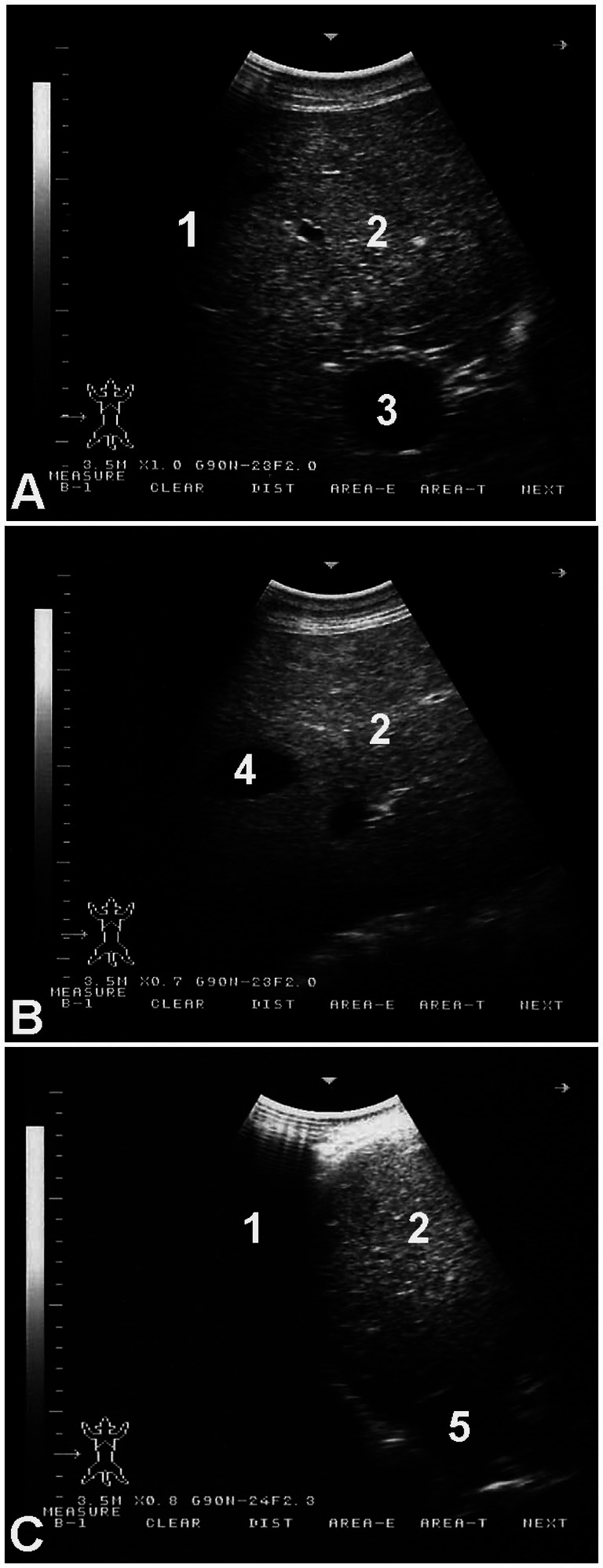
Hepatic ultrasonography in a healthy dromedary camel. The portal vein **(A)** is distinguishable by its echogenic walls and stellate branching pattern. In contrast, the hepatic vein **(B)** appears with anechoic walls. The caudal vena cava **(C)** is identified by its characteristic triangular shape. Labels: 1, lung; 2, liver; 3, portal vein; 4, hepatic vein; 5, caudal vena cava.

**Figure 4:**
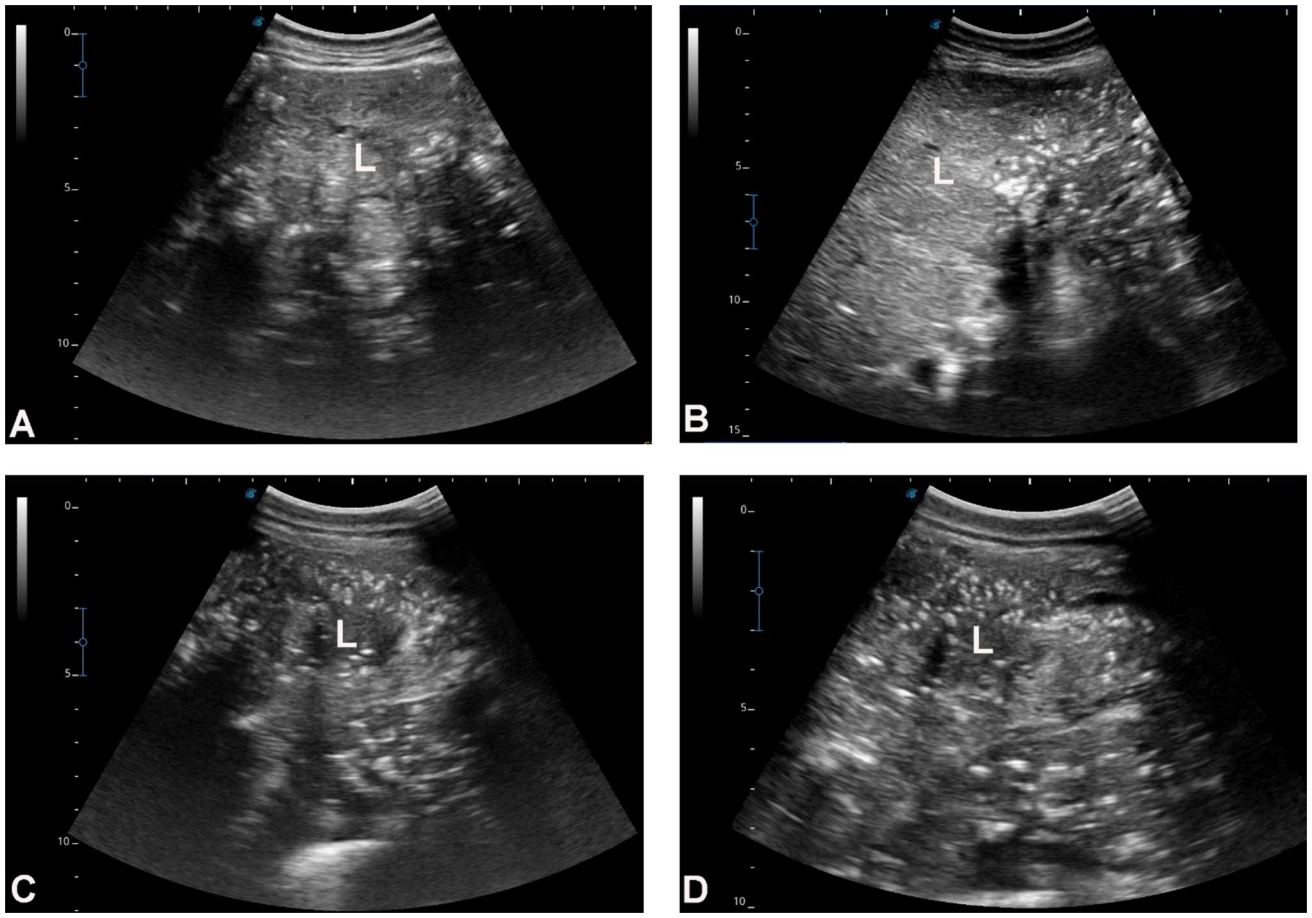
Hepatic ultrasonography in four dromedary camels with liver fibrosis. The hepatic parenchyma exhibits a nodular echotexture and increased echogenicity, which is moderate in images **A,B** and marked in images **C,D**. Hepatic blood vessels, including the portal veins, hepatic veins, and caudal vena cava, are not clearly visualized within the fibrotic liver tissue. Orientation indicators [cranial (Cr), caudal (Cd), dorsal (D), and ventral (V)] are shown in each panel. Scale bars represent 5 cm. L, liver.

**Figure 5:**
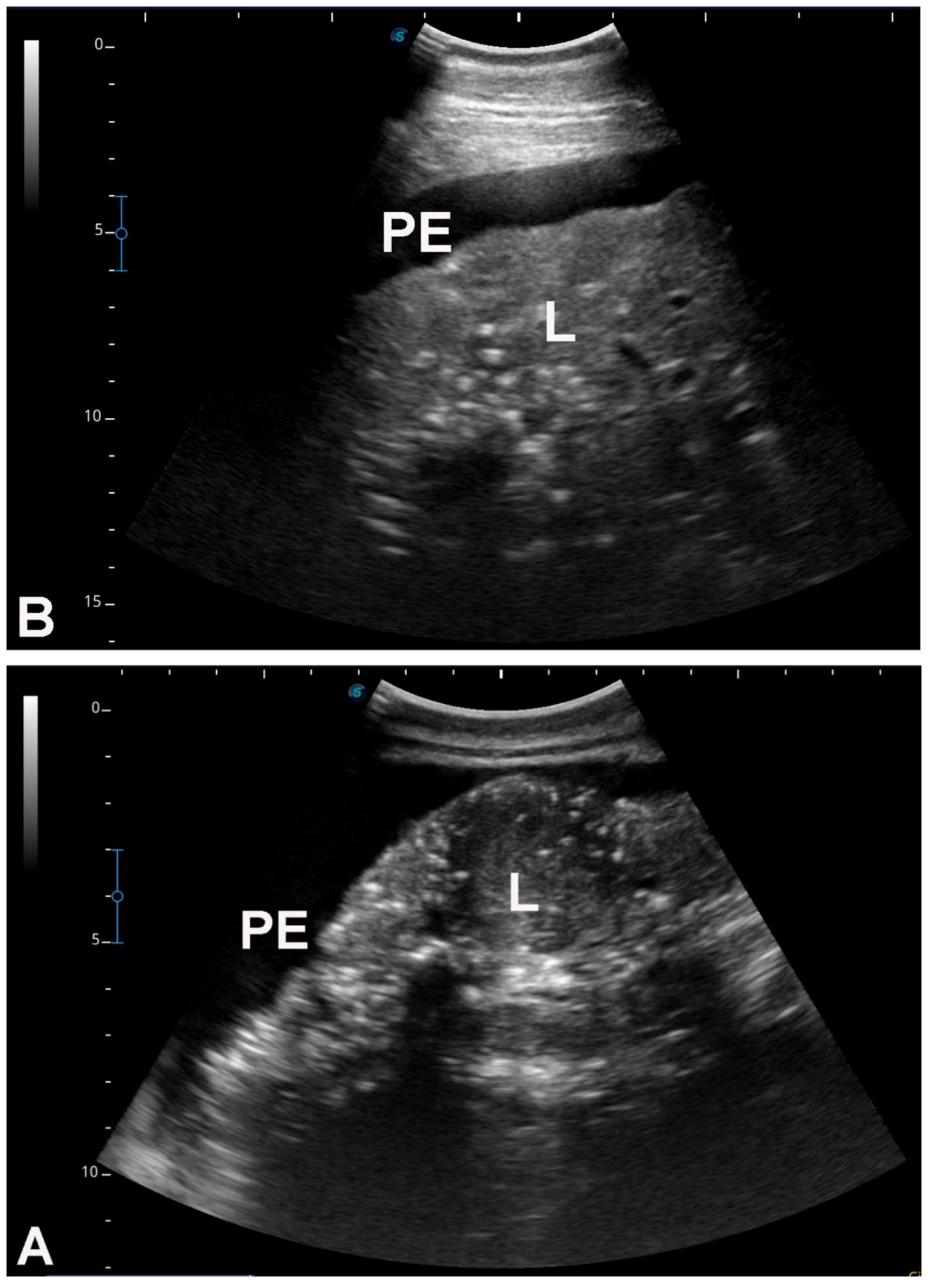
Hepatic ultrasonography in two dromedary camels with liver fibrosis. Nodular lesions are evident throughout the hepatic parenchyma. In both images **(A,B)**, the liver margins appear irregular, and moderate peritoneal effusion (PE) is present. L, liver.

**Figure 6:**
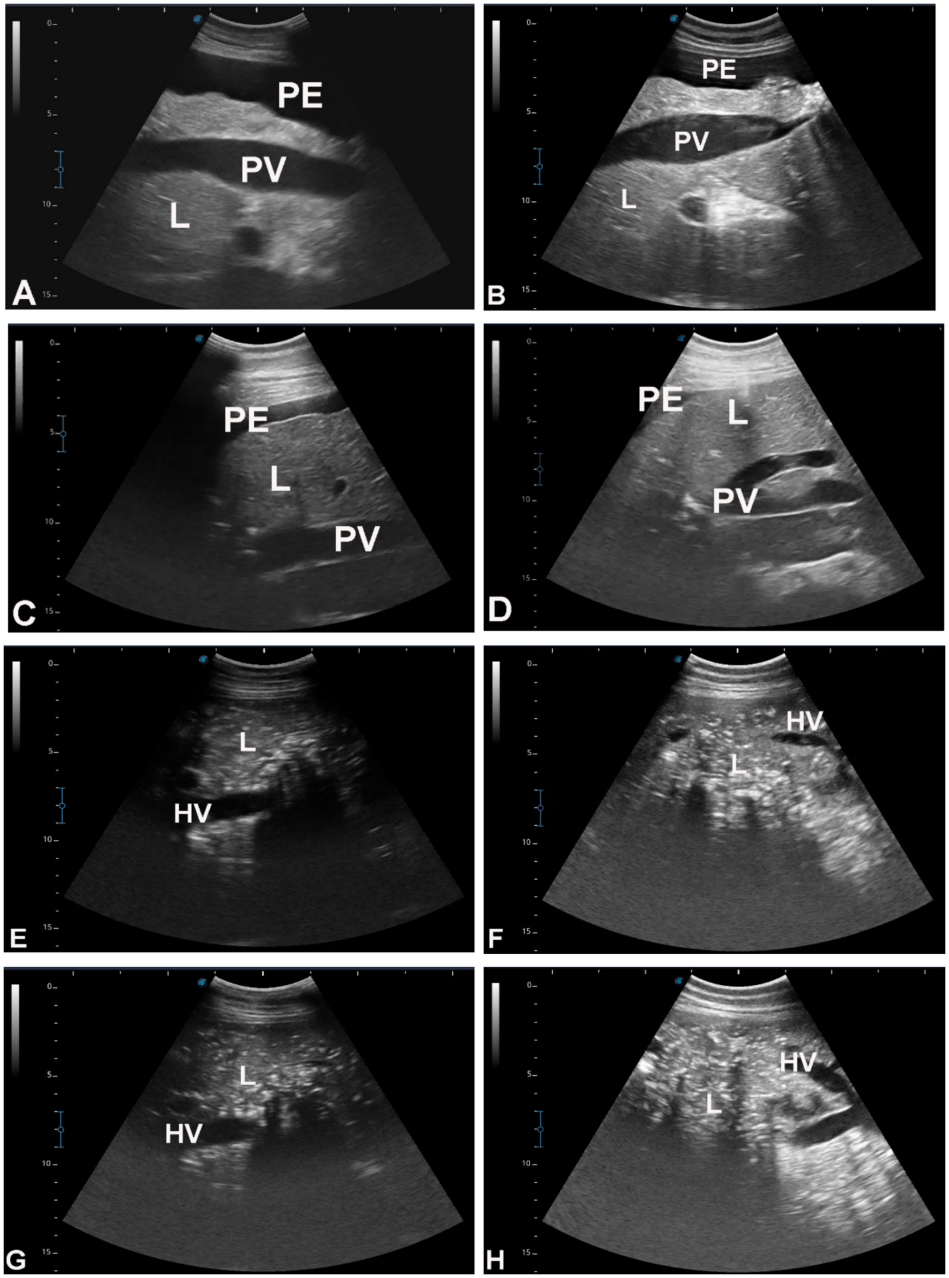
Hepatic ultrasonography in eight dromedary camels with liver fibrosis. In images **A–D**, the portal vein (PV) appears dilated. In images **E–H**, the hepatic vein is visibly constricted. PE, peritoneal effusion; L, liver.

**Figure 7:**
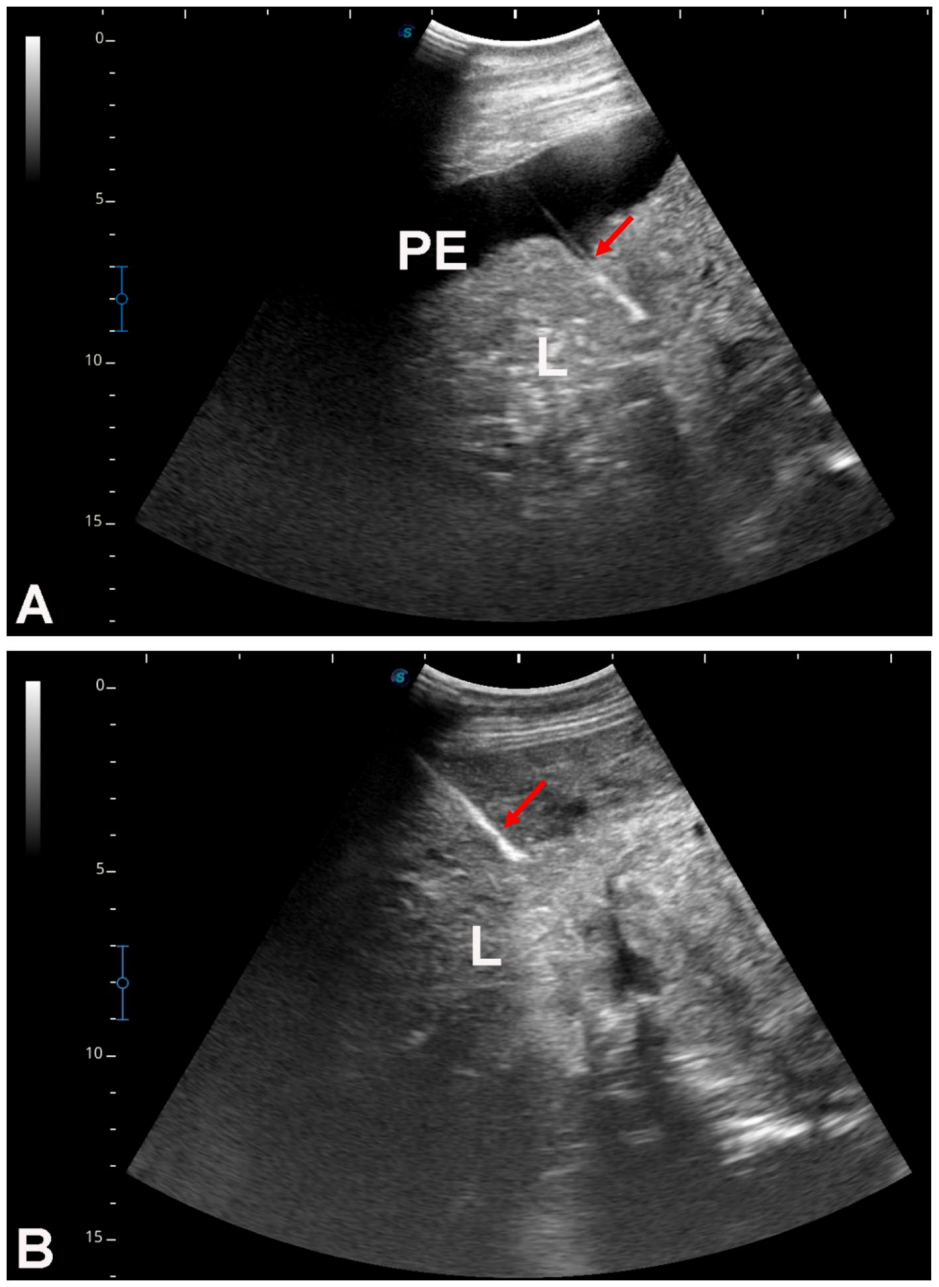
Ultrasound-guided hepatic biopsy in two dromedary camels with liver fibrosis **(A,B)**. The needle path (arrows) is directed toward the center of the hepatic parenchyma. PE, peritoneal effusion; L, liver.

**Figure 8:**
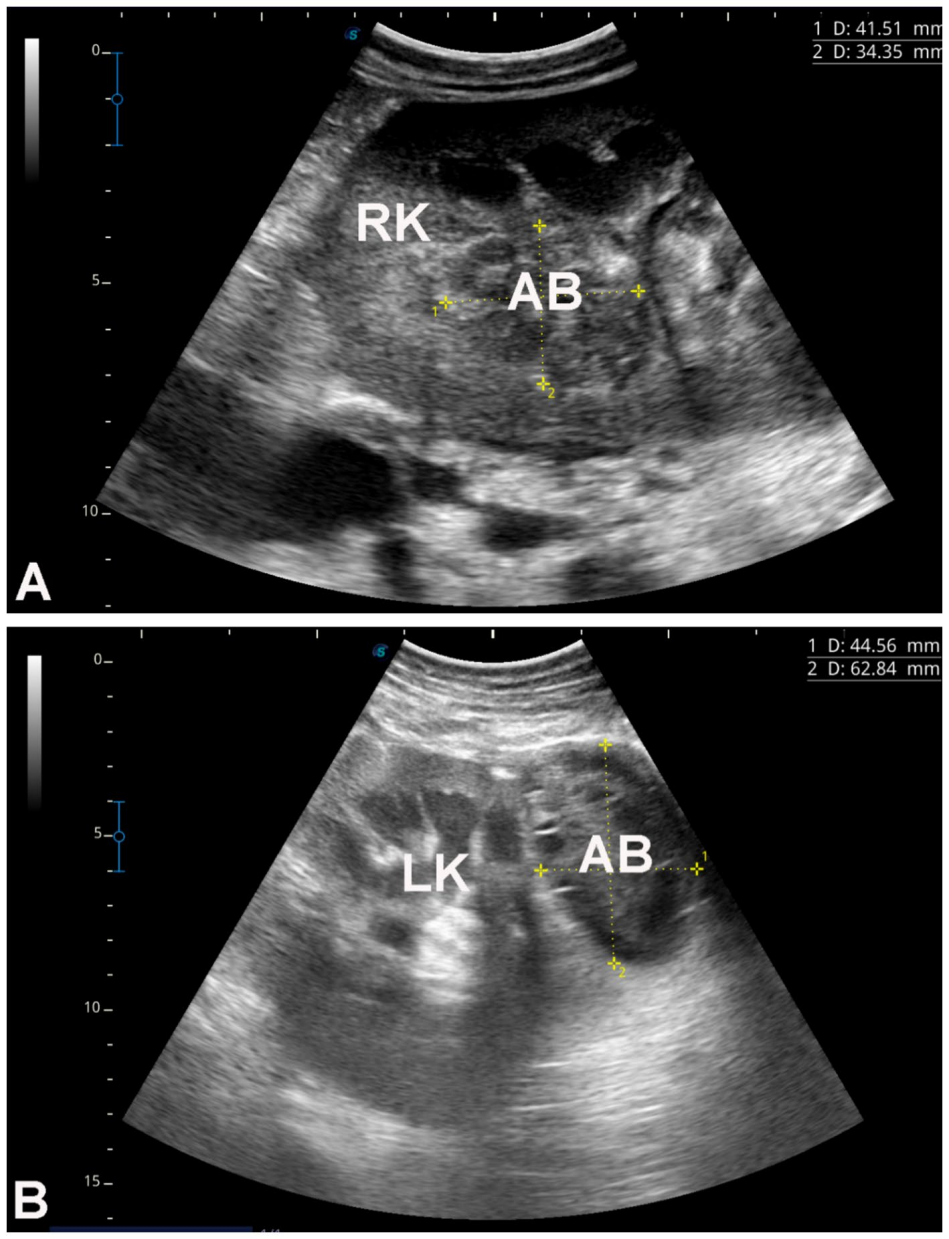
Renal ultrasonography in two dromedary camels with hepatic fibrosis. Image **A** shows an abscess within the right kidney of a camel presented with a one-month history of hematuria. Image **B** shows a perirenal abscess adjacent to the left kidney. Both abscesses were confirmed by ultrasound-guided aspiration of their contents. RK, right kidney; LK, left kidney; AB, abscess.

Figure 3 shows hepatic ultrasonography in a healthy camel, where the portal vein is distinguishable from the hepatic vein. In diseased camels, sonographic examination revealed nodular echo patterns, increased echogenicity, and poorly defined hepatic blood vessels (Figure 4). The liver boundaries were irregular, with moderate peritoneal effusion (Figure 5). In two camels, dilated portal veins were seen, and 4 camels had constricted hepatic veins with peritoneal effusion (Figure 6). Ultrasound-guided biopsy samples were collected from the center of the liver lesion (Figure 7), and abscesses were confirmed in two camels, one with right renal abscessation (Figure 8).

**Figure 9:**
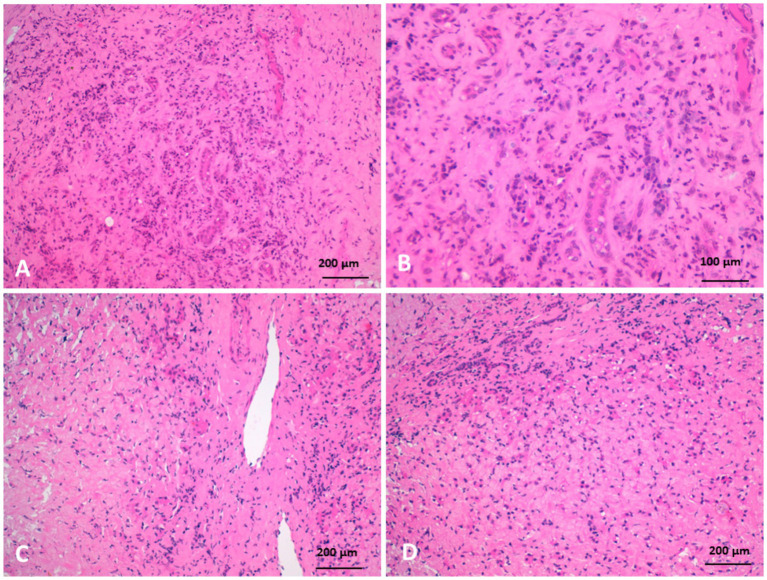
Histopathology of liver biopsies from camels suffering clinically of severe hepatic fibrosis revealed **(A)** severe portal fibrosis, bile duct hyperplasia with newly formed small ductuli, diffuse neutrophilic cell infiltration. **(B)** Is higher magnification of **(A)**. **(C)** Portal fibrosis associated with pressure atrophy of bile ducts. **(D)** Moreover, hepatocytes were atrophied with hyperplasia of Kupffer cells, fibroblastic proliferation, and neutrophilic cell infiltration (H&E).

Histopathological examination of liver biopsies from diseased camels revealed extensive portal fibrosis, marked biliary hyperplasia, and hepatocellular atrophy. Severe portal fibrosis was accompanied by newly formed ductular structures and widespread neutrophilic infiltration. Fibrotic changes in the portal triads—particularly around blood vessels—were associated with pressure-induced atrophy of adjacent bile ducts. Hepatocytes also exhibited atrophy, along with prominent Kupffer cell hyperplasia and fibroblastic proliferation (Figure 9).

**Figure 10:**
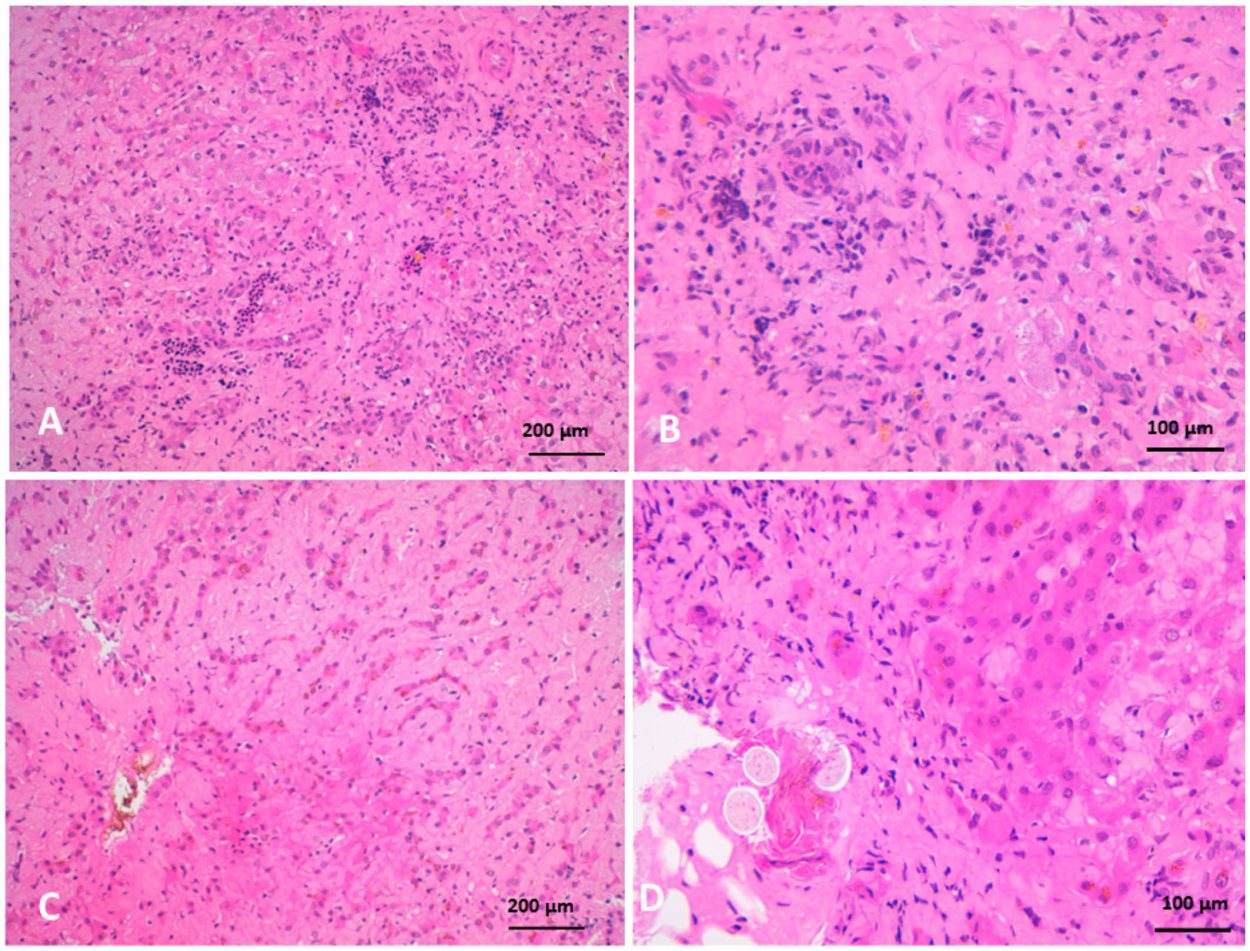
Histopathology of liver biopsies from camels suffering clinically of severe hepatic fibrosis revealed **(A)** biliary hyperplasia associated with severe neutrophilic cell infiltration, extensive fibrosis and widening of portal area that extend to neighboring hepatic tissue, and atrophy of hepatocytes. **(B)** Is higher magnification of (**A**). **(C)** Atrophy of hepatocytes with faint pink homogenous materials accumulated between hepatic cords associated with Kupffer cells hyperplasia containing golden yellow hemosiderin pigments. **(D)** Is a higher magnification of **(C)** (H&E).

**Figure 11:**
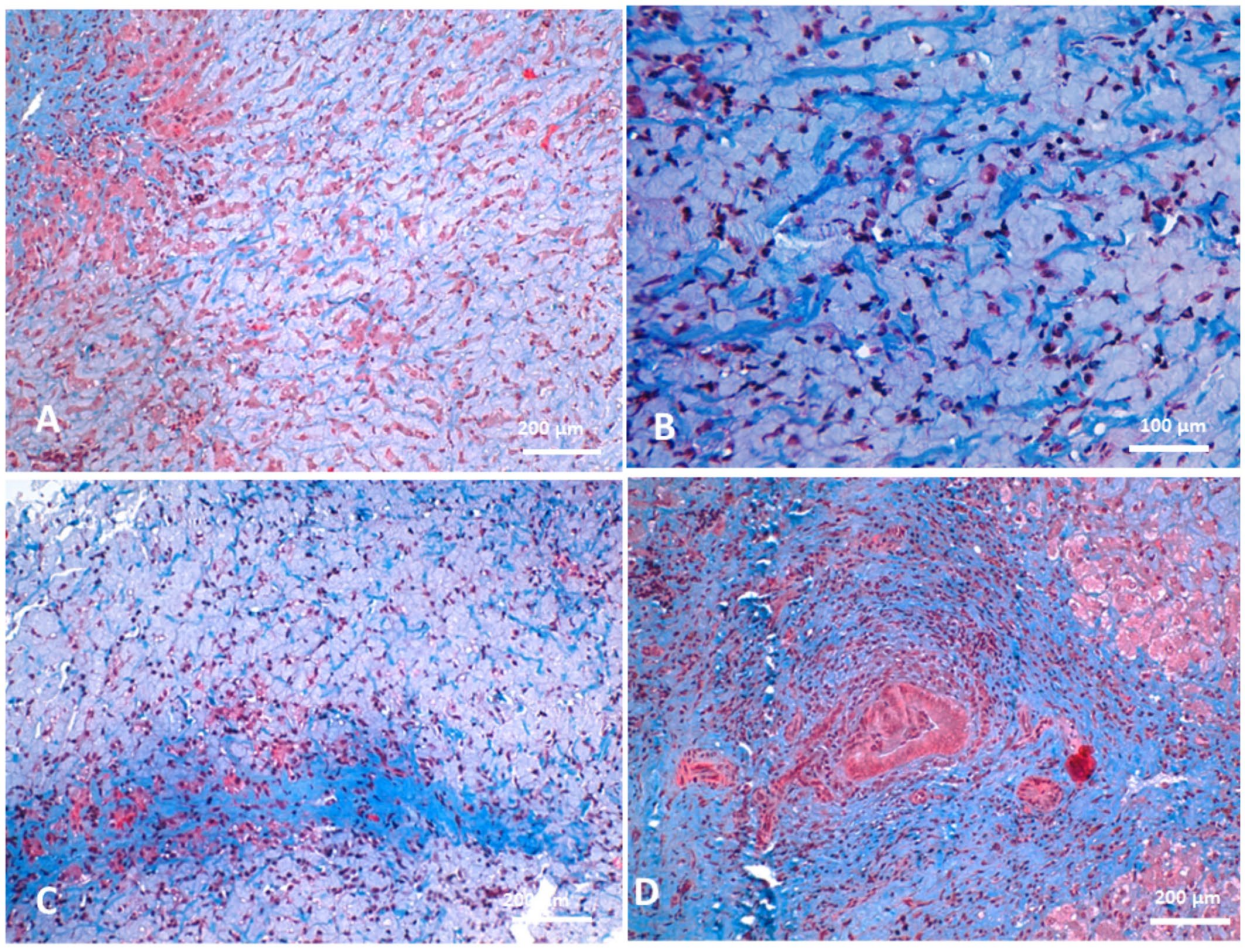
Histopathology of liver biopsies from camels suffering clinically of severe hepatic fibrosis stained with Masson’s trichrome revealed fibrosis in portal triads around portal veins **(A)** and the collagenous fibers extended to permeate between hepatic cords **(B)**. Portal fibrosis might cross hepatic lobules so-called bridging fibrosis in some cases **(C)**. Prominent fibrosis around bile ducts were observed **(D)**.

Biliary hyperplasia was accompanied by severe neutrophilic infiltration, extensive fibrosis, and widening of the portal area, which extended into the surrounding hepatic parenchyma and was associated with hepatocellular atrophy. The portal regions showed vascular and biliary wall thickening, fibroblast hyperplasia, and infiltration by lymphocytes, neutrophils, and pigment-laden macrophages. Hepatocellular atrophy was also observed alongside the accumulation of faint pink, homogeneous material between hepatic cords, as well as Kupffer cell hyperplasia with golden-yellow hemosiderin pigments. The portal areas exhibited neutrophilic infiltration, fibroblastic proliferation, and diffusely hyperplastic Kupffer cells containing hemosiderin. Additionally, a structure morphologically resembling a parasitic egg was observed near the portal region (Figure 10). While this structure was suggestive of a parasitic origin based on its histological appearance, no molecular diagnostics (e.g., PCR) were performed to confirm the species identity. Masson’s trichrome staining highlighted fibrosis within the portal triads surrounding the portal veins, with collagen fibers extending into the hepatic lobules and between hepatocyte cords. The fibrosis bridged adjacent portal areas, indicating bridging fibrosis, and extensive fibrotic encasement of the bile ducts was also prominent (Figure 11).

## 4 Discussion

In camels with hepatic fibrosis, the presenting clinical signs were predominantly non-specific. All affected animals had a history of decreased appetite and progressive loss of body condition. Only 1 out of 16 camels (6.7%) exhibited yellowish mucous membranes. By comparison, in cattle and buffaloes with cirrhotic liver changes due to chronic hepatic fascioliosis, 7 out of 16 animals (43.8%) showed jaundiced mucosa, primarily due to cholestasis caused by obstructive jaundice secondary to fascioliasis (4). In that study, non-specific clinical signs were also common, including poor body condition in 8 animals (50%), low milk production in 7 (43.8%), ventral edema in 7 (43.8%), colic in 4 (25%), and pale mucosa in 8 (50%) (4). In our study, no clinical signs were specific to hepatic disease; however, one camel presented with red urine, and another with deeply pigmented urine.

Ultrasonography has proven to be an essential diagnostic tool in dromedary camel medicine for evaluating thoracic and abdominal organs and diagnosing various intra-abdominal disorders as well as for reproductive disorders (11, 16, 19–31). Moreover, ultrasound has shown utility in the *ante mortem* diagnosis of abdominal masses in camels (32, 33).

Liver biopsy is considered the gold standard for diagnosing hepatic diseases in veterinary medicine, particularly in companion animals and large species, including camels and other ruminants (13, 34). It provides essential histopathological insights that are critical for identifying the type and severity of hepatic lesions, guiding prognosis, and informing therapeutic decisions (35). While non-invasive diagnostics such as serum biochemistry, ultrasonography, and serologic testing are valuable in identifying liver dysfunction, they often lack specificity, and biopsy remains indispensable in cases with ambiguous or overlapping findings (36). In cases of hepatic fibrosis, biopsy enables direct assessment of architectural changes in the liver parenchyma and biliary system, making it especially valuable in chronic hepatic conditions (37).

In camels, hepatic fibrosis may be underdiagnosed due to the limited use of biopsy in clinical settings. Most veterinary clinics in the Middle East rely on clinical examination and, occasionally, serum hepatic function tests, which may be misleading if influenced by muscle catabolism or other illnesses. In a slaughterhouse study of 156 apparently healthy camels, liver atrophy and fibrosis were identified in 1.28% of cases (38). Similarly, in Egypt, 688 camel livers examined post-slaughter revealed histologically confirmed severe necrosis with fibrosis and biliary duct hyperplasia in only seven cases (1%) (39). In India, hepatic cirrhosis was found in 8.75% of 80 camels examined in a slaughterhouse (40). These variable figures suggest that the true prevalence of hepatic fibrosis may be underrepresented. In the current study, 16 cases of liver fibrosis were diagnosed clinically and confirmed through liver biopsy, underscoring the potential value of broader application of this diagnostic technique for more accurate prevalence estimates.

Histopathological findings in the affected camels revealed extensive fibrosis, bile duct hyperplasia, and hepatocellular atrophy. Severe portal fibrosis with proliferation of newly formed ductules and diffuse neutrophilic infiltration were noted. Fibrosis in the portal triads, associated with blood vessels, resulted in pressure atrophy of bile ducts. Additionally, Kupffer cell hyperplasia and fibroblastic proliferation were observed, hallmark features of hepatic fibrosis. In cattle, hepatic fibrosis is frequently caused by *Fasciola* spp. infestation. Migrating metacercariae create visible dark red tracks in the liver parenchyma, inducing hemorrhage, necrosis, and granulation tissue formation that progresses to cirrhosis (41, 42). More recently, fatty liver has been implicated in liver fibrosis in dairy cattle through mechanisms involving oxidative stress, hepatic apoptosis, and inflammation (5). In dogs, hepatic fibrosis typically follows chronic hepatitis (37). In this study, parasitic involvement could not be definitively excluded, as several camels exhibited eosinophilia and portal area egg-like structures. Neutrophilia and monocytosis were common hematological findings, and histopathology revealed neutrophilic infiltration and biliary fibrosis, consistent with chronic active hepatitis and cholangitis.

## 5 Conclusion

This study presents the first integrated clinical, biochemical, ultrasonographic, and histopathological characterization of hepatic fibrosis in dromedary camels. Chronic, non-specific clinical signs correlated with hematobiochemical markers of systemic inflammation and hepatic dysfunction. Ultrasonographic abnormalities closely reflected histopathological changes, supporting the utility of imaging in diagnosis. The identification of parasitic structures in some cases suggests possible parasitic infections as a contributing etiological factor. Together, these results emphasize the complexity of hepatic fibrosis and the need for comprehensive diagnostic strategies to enable early detection and effective management in dromedaries.

## Data Availability

The raw data supporting the conclusions of this article will be made available by the authors, without undue reservation.
